# South Asian Women′s Perspectives and Recommendations on Gestational Diabetes Care and Postpartum Type 2 Diabetes Prevention in Aotearoa New Zealand: A Qualitative Study

**DOI:** 10.1155/jdr/8712576

**Published:** 2026-09-24

**Authors:** Sumera Saeed Akhtar, Sharon Leitch

**Affiliations:** ^1^ Department of Primary Health Care, Dunedin, Faculty of Medicine, University of Otago, Dunedin, New Zealand, otago.ac.nz

**Keywords:** early onset of Type 2 diabetes, gestational diabetes, postpartum follow-up, prevention, South Asian women, Type 2 diabetes

## Abstract

South Asian (SA) women in New Zealand face a disproportionately high risk of developing gestational diabetes mellitus (GDM) and subsequent Type 2 diabetes (T2D). Despite this elevated risk, significant gaps exist in culturally appropriate care for GDM and long‐term postpartum follow‐up. This qualitative study explored the experiences of SA women to identify the gaps and barriers encountered during and after GDM in transitioning from antenatal GDM care to postpartum primary healthcare and seek suggestions for improvement. To inform culturally responsive care strategies that can reduce the risk of progression to T2D after GDM, 30 semi‐structured in‐depth interviews were conducted with SA women diagnosed with GDM within the past 5 years. Participants were recruited through purposive sampling across New Zealand. Interviews were transcribed verbatim and analysed using an inductive thematic approach. Six key themes with sub‐themes were developed: (1) awareness and education; (2) culturally tailored resources; (3) community support; (4) moving backwards to an unhealthy lifestyle; (5) preventing T2D; and (6) improving GDM and postpartum care. Participants advocated for prepregnancy or early counselling, community‐based education to reduce stigma, culturally relevant dietary guidance, subsidised follow‐up healthcare providers and structured long‐term postpartum follow‐up. The emotional burden of GDM and inconsistent healthcare communication were also prominent concerns. The findings highlight the need for integrated, culturally congruent and continuous care models for SA and ethnicities at high risk during and after GDM. Incorporating women′s suggestions based on their lived experiences into service design may improve postpartum health outcomes and reduce future risk of GDM and T2D.

## 1. Introduction

The worldwide public health burden of type 2 diabetes (T2D) continues to rise [1], with a shift towards onset among younger individuals [2]. One of the risk factors for T2D is diabetes during pregnancy, known as gestational diabetes mellitus (GDM), which is estimated to affect 2%–9% of all pregnancies [3]. GDM elevates the risk of progression to T2D tenfold [4]. Many women develop T2D within 6 years of a GDM diagnosis, often while still of reproductive age [5]. Pregestational T2D increases the risk of adverse perinatal outcomes in subsequent pregnancies. Furthermore, a longer duration of T2D increases the risk of developing diabetic macrovascular and microvascular complications [6]. Risk factors for GDM include a history of GDM, a large for gestational age baby, a family history of T2D, high prepregnancy BMI, increasing maternal age, obesity and the migration of women of high‐risk ethnicities to western countries for better opportunities [7]. In the 2023 Census, 17.3% of the NZ population identified as Asian, with South Asian people from Afghanistan, Bangladesh, Bhutan, India, the Maldives, Nepal, Pakistan and Sri Lanka being the fastest‐growing subgroup [8], comprising 6% of the New Zealand (NZ) population [9].SA women are almost four times more likely to develop GDM than non‐SA women in NZ [10], with nearly one in three pregnancies affected. GDM and T2D present a substantial burden of disease for South Asian women. GDM is often linked to adverse outcomes for mother and baby, including preeclampsia, C‐section, macrosomia, neonatal hypoglycaemia, excessive foetal growth and birth trauma, as well as the later development of T2D and other metabolic conditions [11, 12].

Most health systems provide structured care during GDM but lack structured post‐GDM support and follow‐up in the immediate and long term. For women diagnosed with GDM, the shift from postpartum to interpregnancy or general well‐woman care is often poorly executed, leaving many women with GDM feeling abandoned by the health care system after delivery [13, 14]. They report receiving limited support and a lack of follow‐up for information and monitoring; therefore, they do not realise the need to maintain a healthy lifestyle to avoid T2D [15–17]. As immigration increases worldwide, the incidence of GDM and related conditions such as early onset of T2D is expected to increase as well. Health professionals may struggle to understand the needs of an increasingly diverse population. Likewise, migrant ethnicities face challenges in adapting their cultural preferences to the healthcare available in their new country [18].

This study is primarily aimed at identifying the gaps and barriers encountered during and after GDM in transitioning from antenatal GDM care to postpartum primary healthcare and current lifestyle by exploring the lived experiences of South Asian women with previous GDM. Additionally, it seeks to gather participants′ suggestions for improving postpartum care after GDM to prevent the early onset of T2D. This contributes to the existing literature and can support the development of future interventions to reduce disparities among ethnic minorities. This is especially important for high‐risk groups (e.g., South Asian, Pacific and Māori women) who face a disproportionate burden of GDM and its complications; it may also help to alleviate some of these inequities.

## 2. Method

### 2.1. Ethics Approval Statement

This study was approved by the University of Otago Human Ethics Committee (Reference: 19/004).

### 2.2. Study Design

Semi‐structured in‐depth interviews were conducted to explore and understand SA women′s lived experiences, reflections and suggestions for improving GDM management and postpartum care [19].

### 2.3. Participant Recruitment

Purposive sampling was used to ensure representation of SA women of different nationalities [20]. Inclusion criteria were SA women who self‐identified by country of birth (Afghanistan, Bangladesh, India, Nepal, Pakistan or Sri Lanka), were aged 18 years and above, had been diagnosed with GDM within the last 5 years and could communicate in Hindi, Urdu or English. Participants also had to be permanent residents or NZ citizens to ensure eligibility for publicly funded healthcare services. Participants were recruited across NZ by advertising in public places, on social media pages and to SA communities and organisations. Interested women self‐identified in response to the study advertisement and contacted the first author (S.S.A.) directly to express their interest in participating. S.S.A. had no prior relationship with participants.

### 2.4. Data Collection

Before commencing the interviews, participants were informed that S.S.A. is a pharmacist and health professional researcher. Participants were also informed of the aims of the study. We conducted interviews from April to July 2024. After initial contact, the first author, S.S.A., verbally explained the study to participants in their preferred language (English, Hindi or Urdu). Because most participants could read and understand English, we provided the information sheet and consent form in English through an online Qualtrics link. Participants provided informed consent before accessing the brief demographic survey.

S.S.A. conducted all telephone interviews at a time convenient to participants. There were no repeat interviews. The semi‐structured interview guide included probes on knowledge, awareness and GDM management, as well as post‐GDM life. Interviews lasted 45–80 min and were audio‐recorded with consent. S.S.A. conducted 30 interviews: 26 with SA women in English and four in Hindi/Urdu; S.S.A. transcribed and translated the four Hindi/Urdu interviews verbatim into English. S.S.A. also transcribed the English interviews verbatim. Recruitment continued until no new themes were identified from data analysis [21]. Each participant received a $50 supermarket voucher after the interview.

### 2.5. Data Analysis

Participants were allocated a unique study ID number. All identifying information was removed from the transcriptions, including names and ethnicity. We chose an inductive thematic approach because the study is aimed at exploring participants′ perspectives and suggestions based on their lived experiences without a predefined coding framework. This method allowed codes and themes to emerge directly from the interview data, keeping the analysis closely aligned with participants′ accounts [22]. S.S.A. read and reread the transcripts and coded them using an inductive thematic approach [23, 24] with NVivo software. Codes were organised into categories and sub‐themes, which the authors (S.S.A. and S.L.) discussed to develop concepts. The authors continued the iterative process of coding and interpreting data until they reached consensus. S.S.A. is a female researcher and a health professional (pharmacist) of SA ethnicity and is aware of the potential influence of this on the study. S.S.A. strove for reflexivity by acknowledging her background and positionality, considering how these might affect those with opposing beliefs while keeping a journal to document reflections throughout the research process. S.L. is a female NZ European general practitioner (GP) working in clinical practice and academia in NZ. S.L. acted as a reflexive prompt by identifying and querying cultural assumptions in the study findings. Participants were invited to provide feedback on the findings; however, none responded. The COREQ checklist [25] was used to guide reporting.

## 3. Results

Participants were 30 SA women diagnosed with GDM, aged between 24 and 46 years (median age, 36 years) (Table 1). Most women reported normal blood glucose levels postpartum, though nearly a third were uncertain of their status, and two were diagnosed with T2D within 2 years. Although 70% received advice from healthcare professionals during pregnancy on preventing T2D, only 53% reported trying to make lifestyle changes, primarily focusing on diet, sugar reduction, and, in some cases, medication.

**Table 1:** Demographic characteristics of participants.

		*N*	In %
Country of birth	Afghanistan	3	10
	Bangladesh	4	13
	India	11	37
	Nepal	3	10
	Pakistan	6	20
	Sri Lanka	3	10
Age range	24–46 years	Median 36 years	
Number of years in New Zealand (range)	5–24 years	Median 10 years	
Number of pregnancies in which GDM is diagnosed	1	23	77
	2	4	13
	3	3	10
Are your blood glucose levels normal now?	Yes	19	63
	No (diabetes)	2	7
	Do not know	9	30
Did your health professional talk to you about how to prevent diabetes in later life during GDM management?	Yes	21	70
	No	9	30
Have you implemented any long‐term changes in your lifestyle as a result of the diagnosis of GDM?	Diet + exercise	4	13
	Diet + less sugar	5	17
	Diet + less sugar + medicine	7	23
	No change	14	47

From the interviews, six major themes were identified: awareness and education, culturally appropriate resources, community support, moving backwards to an unhealthy lifestyle, preventing T2D and improving GDM and postpartum care. Table 2 summarises the quotations by theme.

**Table 2:** Reflections on long‐term implications and future pregnancies.

Themes and sub‐themes	Verbatim quotes
Awareness and education	In 10: ‘My GP, especially because he knew my family history and that we all have the same GP. And to be honest, because I have PCOS in my head, I just thought it′d be really difficult to get pregnant at first.’
	In 5: ‘If I were informed early in pregnancy that I am at high risk, I might have controlled my diet and increased my physical activity. Just like after a caesarean, they told you that you are at high risk of blood clotting, so you have to take blood thinners and do walks as movement is compulsory, they told me. So, I was very careful.’
	In 15: ‘Obviously, I was upset. I did not know about it, and I have not had previous education about it that happened during the pregnancy, if I were aware, at the start of the pregnancy, that GD would come in for people like, for example, my mother, she has diabetes, and I was not taking care in terms of food, in terms of anything because I did not know about it. It is gestational diabetes. However, in my third pregnancy, I was aware of it from the start.’
	In 18: ‘And not just mothers, other people who need to be involved as well, like, you know, husbands or the other family members, caretaker, whoever is around you, otherwise, it′s difficult to do it on your own. I think they need to be aware as well. Similarly, as the awareness has been provided to mothers, I think the same level of awareness should be given to them. Yeah, it should not be like, you know, sometimes what happens is I′m being strong, OK, I′ll not eat it. The family might say that′s OK, just once. Please eat it. Eat. Yes, it makes a lot of difference.’
Culturally appropriate resources	In 6: ‘Non‐vegetarians can eat meat at night and avoid carbohydrates. They even told me to eat eggs. So, I started eating because that was helpful. I would recommend that there be a diet plan for vegetarians, so that if they are non‐vegetarians, it′s easy for them to manage gestational diabetes. But if you are a vegetarian, then it is not easy.’
	In 29: ‘How we eat in our culture, like Indian and Nepali. There is not much information, especially for us, like how we eat, how much and what kind of food. If there is much more information like this, it would be helpful.’
	In 20: ‘When you get it the very first time, you have no clue what you can eat and what you cannot eat. Of course, we go with the usual flow of what we are used to. For me, rice, curry and white bread were part of meal. But I think even you will get diet advice. It will be good for them to give you a blue booklet of what is good for you to eat and things, so that you know you can. At least have an example of. OK what you can eat and what you cannot.’
Community support	In 29: ‘I think our local Asian communities have a crucial role to play here by spreading awareness and education.’
	In 23: ‘Not everything can be done by the healthcare system; they are already doing a lot; community organisations can share some of the responsibilities by providing support to isolated young mothers.’
	In 30: ‘I think there is a lot of community education needed. There are a lot of misconceptions and myths within our community. They go saying Oh, make sure you do not get GD on your 26th week. Do not eat any sugar for a week before you test, and I′m like all you see does not result in. And after they got diagnosed with GD, they suggested to each other, eat whatever you want, it′s your time.’
	In 10: ‘I think there is an element of shame and stigma that gets associated, that people do not want to take it seriously, and even talk about it, and advise other women to ignore the diagnosis and do and eat whatever they like.
Moving backwards to an unhealthy lifestyle	In 18: ‘Because the importance changes, right? Once you have the little one and even after the checks by the healthcare professionals, everything is focused on the child after your delivery.’
	In 20: ‘I think we, especially South Asian women, do not care. I do not have any other responsibilities. Yes, it′s just me, my husband, my four kids, and we also run our own restaurant. He works way more hours than he would if he were working for others.’
	In 30: ‘You do not have time to plan your meals or go to the gym, as you cannot even finish all the household chores. Your life changes drastically after having a baby.’
	In 13: ‘I think the first one was a bit easier to manage GD than the second because I had to take care of my older one, which made it more challenging. I found it a lot harder to keep up with my exercise this time.’
Preventing Type 2 diabetes	In 22: ‘I was very much depressed. Like, I do not want my kids to suffer from diabetes because I have seen my parents; it′s been a long time since diabetes, and they are suffering from neuropathy. So, I just do not want diabetes. I was very affected. I need more guidelines to maintain a healthy lifestyle to avoid diabetes for my children.’
	In 10: ‘My aim is obviously not to get diabetes, and if I need to make a conscious decision, my daughter also does not get it.’
	In 13: ‘I′ll have to try really hard. Yeah. I mean, yeah, that′s the thing. When I′m really, really very careful about my diet for a long time and when I′m doing my exercise regularly for a few months, that′s when I start losing some weight also. And that′s when I am decreasing my HbA1c as well, so yeah if I get off track a little bit then I′ve lost all the progress.’
	In 9: ‘I should keep in mind that in later life I should try not have diabetes. I should just to exercise and reduce carbs. And then, I think just try to be healthy and then to take all the precautions.’
	In 29: ‘I think I should, and then I think it′s a wake‐up call for me, and I will take care of myself in terms of these things. And then the hospital should, because they have every record. They should increase the resources. They should follow up every 3 years or something like that. Keeping up like and you had this and then if you can give this template that it has that you should exercise, you should do this, and then this is the food you should avoid, and this is the kind of food you eat and the diet you should follow. A dietitian may be helpful. Just follow‐up. That would be like because once I have this that will be another story. I think preventive action they can take. Just to educate women about it.’
Improving GDM and postpartum care	
Structured regular follow‐up	In 9: ‘I think they should follow up, since I had this since then, even I did not get any information from hospital or from GP.’
	In 24: ‘GP visits should also be subsidised, as we often become lazy due to the cost, since we have to pay the GP fee every time. Even when we request online prescriptions or test repeats, there is a charge.’
	In 12: ‘Yeah, it should be regular. It will be good and it will give us also some control, like, OK, I have to go to the GP to get the blood test and make sure to get the sugar under control, because if the result is not good, we again have to go back to all those medicines and treatments.’
	In 14: ‘But at least maybe in a few months. They could get in touch and ask for a blood test because I was told to get it at 3 months, and then maybe in 1 year. But there is a big gap between them. So, I think that every 3 months, testing and keeping in touch would be very beneficial for us, so we do not revert to an unhealthy lifestyle.’
	In 20: ‘No, I have not, nothing comes up. I have not visited my GP in over a year, but I have not received any calls or notifications. Yeah, can I go and request a test?’
	In 28: ‘Yeah, maybe. Only GP asked me once. I think it was after 8 months or 6 months after delivery that they asked me to check my blood sugar, and it was fine. Then, after that, nobody asked me to do it again, so I came back and got the blood sugar level test done and all.’
Subsidised consultation with a dietitian/nutritionist	In 21: ‘It would be good to see dietitians or nutritionists. That will be helpful. So this kind of thing, as a pregnant woman with GDM, goes to the diabetes clinic and if they have some offer in the future for this, like postpartum things. If they could provide that knowledge, that would be great.’
	In 26: ‘Some meal books or something like how to make meals for vegetarians, especially during GD management or postpartum.’
	In 4: ‘Yes, I would like to consult a dietitian, as I had previously wanted to see one, but the cost was too high for me to afford. GP visits should also be subsidised, as we often become lazy due to the cost, since we have to pay the GP fee every time. Even when we request online prescriptions or test repeats, there is a charge.’
Access to advanced continues glucose monitoring tools	In 19: ‘No, actually, when I was taking insulin, I used to get frustrated because it was a bit painful. So, I used to get bruises as well. That was a bit frustrating, you know. I used a sensor, and I recommend it be subsidised, because it was a bit easier; I do not have to prick my fingers repeatedly.’
	In 30: ‘Glucometer readings were not always accurate as sometimes, if you have not sanitised your pricking finger, your reading will be wrong, and my glucometer stopped working and had to go to clinic to replace it, why not give Dexcom? I am sure it will be better in every aspect.’
Addressing the emotional burden	In 22: ‘It′s really hard. It is a must, like we do not have help, you know, the depression. That′s where the ladies, the women, mothers, are getting depressed because they do not have proper support either from the family or the government. They need to consider this aspect. I′m very lucky in this aspect because my husband is always with me. And he has to quit his job; everything is for me and the kids. He takes care of us 24/7. So I′m lucky. But if the husbands are really working. And mothers are staying alone with their kids. Obviously, she has been struggling a lot without any support, it′s not possible.’
	In 3: ‘I mean, there are various things. I suppose the thing is that there are things you want to do, but yeah, it depends on how much help you get. And you know, if you have postnatal depression, then that′s a barrier as well, because even though you would not try to do something. You know, psychologically, you just cannot control yourself, and you can do something bad.’
Green prescription	In 8: ‘But in my situation, going to the gym is so hard, because of the head covering, then the dress code. Then, we do not have any ladies‐only gyms because all of them are mixed. That′s why I prefer not to go to the gym. However, if a gym is available like that, it′s extremely helpful. Yeah, women‐oriented, that′s so helpful. And the other thing, Zumba classes, again, I think everything′s mixed up. Yeah, only that one is making me go for the walk.’
	In 18: ‘I would say, like you know, rather than moving pools in the very first year they would need someone who can give them some exercise which they can follow at home. But what′s right for them at that stage. Because not every exercise is right, like you know with the. With the delivery process they go through, increased support in those terms can be very helpful.’
	In 4: ‘Subsidised activities, much like a green prescription, should include activities specifically for women. The council pools should have women‐only days, at least 1 or 2 days a week. Similarly, they have gyms or leisure centres that should be ladies‐only; this initiative has started in some places, but it is still limited, so not everyone has access to them. There should also be information in Urdu.’
Healthcare equity	In 30: ‘Initially, I was very confused as I had experienced different care in different cities of New Zealand during my two pregnancies. I believe there should be the same kind of healthcare services available nationwide for everyone. I was confused.’
	In 21: ‘My friend, who was also diagnosed with GD, had a different experience in different regions. Some things were not done to me, but they had been done to my friend. I never get to see a dietitian, but she did. There was a gap, and I told my midwife that it needed to be covered.’
	In 26: ‘The midwife for my friend tested her for a cholesterol level. But my midwife did not ask me to get that test. My friend and I had deliveries 2 weeks apart, but in the end we had our babies on the same day. There was a lack of guidance from midwife. When I told her about it, she told me that the pregnancy diabetes centre should tell me about it. When I asked them, they said midwife should do it. But, I did advise them to let other people know it′s important.’

### 3.1. Awareness and Education

Many women expressed a desire for early pregnancy or preconception counselling from their GP, particularly if they have additional known risk factors for GDM, in addition to SA ethnicity (such as family history, polycystic ovary syndrome (PCOS), obesity or higher body mass index [BMI]). Counselling could set expectations and improve their understanding of pregnancy risk. Encouraging early lifestyle changes may prevent or mitigate GDM severity, particularly when implemented well before the standard screening period (24–28 weeks of gestation).

Most women believed that clear written and visual resources explaining the nature of GDM, self‐management and the rationale behind treatment options would be very helpful. Women wanted realistic strategies for self‐management post‐GDM, as well as information about future T2DM risk for themselves and their baby. Some women believed that this would help them manage weight gain during pregnancy and lose weight after pregnancy.

Participants felt that a family‐inclusive approach, which included their husbands and in‐laws, was crucial. Increased family involvement in learning about GDM management can help motivation and improve adherence for healthier lifestyle changes (for example, by following healthy diets, grocery shopping or preparing appropriate meals). This support makes it easier for SA women to adhere to dietary and exercise recommendations to manage GDM.

In 13: ‘So, with my first one, I had my in‐laws here for a while, but then I think that what happens is you have to educate them as well. What is a good diet, what do you need and all that.’

### 3.2. Culturally Appropriate Resources

Participants believed that clear resources explaining the nature of GDM, self‐management and the rationale behind treatment options would be very helpful. Women want practical strategies for self‐management post‐GDM, as well as information about future T2DM risk for themselves and their baby. Some women believed that this would help them manage weight gain during pregnancy.

In 4: ‘There should be a forum for people with diabetes or prediabetes, especially culturally addressed. There will also be sessions on awareness and information, which are easily available and accessible, or can be accessed online. There should also be information in Urdu.’

Participants want dietary educational materials and advice to be tailored to reflect the foods, cooking methods and recipes commonly consumed within SA communities. Recipes need to be easily manageable during pregnancy and with children. Participants want vegetarian meal plans that meet their nutritional needs during and after pregnancy. Specific advice was needed on how to modify traditional recipes to make them healthy, as most vegetables and foods traditionally used in SA cooking are unavailable in NZ. Guidance is needed on how to reduce the carbohydrate load in roti, rice and curries. Although some dietitians did accommodate these requests and provide general advice, several women wanted a more detailed, recipe‐based guide. A few participants suggested greater involvement of SA dietitians/nutritionists could provide more relevant information for SA women, but most participants believe that the ethnicity of the dietitian does not matter as long as the information is relevant.

In 29: ‘How we eat in our culture, like Indian and Nepali. There is not much information, especially for us, like how we eat, how much and what kind of food. If there is much more information like this, it would be helpful.’

### 3.3. Community Support

Participants identified a role for community organisations to encourage young families to adopt and support healthier eating habits through family education and support sessions.

In 30: ‘I think there is a lot of community education needed. There are a lot of misconceptions and myths within our community.’

In 29: ‘I believe our local Asian communities have a crucial role to play in spreading awareness and education.’

Participants suggested considering community initiatives that respect cultural sensitivities and support busy mothers in maintaining physical activity, such as subsidised women‐only exercise classes, yoga sessions or walking clubs. Counselling services and peer support groups could assist women in managing the emotional distress and burden of self‐monitoring associated with GDM.

In 23: ‘Not everything can be done by the healthcare system; they are already doing a lot; community organisations can share some of the responsibilities by providing support to isolated young mothers.’

Participants mentioned that GDM is associated with shame and stigma. They suggested that education for the SA community could help reduce shame, cultural stigma, misconceptions and myths about GDM. This could further encourage adherence to management plans, peer support and ultimately lead to improved outcomes.

In 10: ‘I think there is an element of shame and stigma. That gets associated with people not wanting to take it seriously and even talking about it and advising other women to ignore the diagnosis and do and eat whatever they like.’

### 3.4. Moving Backwards to an Unhealthy Lifestyle

Participants described the difficulty of sustaining the rigid dietary regimen and exercise routines imposed during pregnancy. Some participants tried to maintain healthy lifestyle changes postpartum. Some participants experienced a sense of relief when their blood sugar levels ‘returned to normal’ after delivery. However, some were diagnosed with insulin resistance or prediabetes, and two had been diagnosed with T2D within 2 years (Table 1).

Caring for the baby, busy family lives, work responsibilities and the cultural emphasis on eating an abundance of food during postpartum (especially from mothers or mothers‐in‐law) were cited as barriers to maintaining healthy habits.

In 14: ‘Extra barriers include having many responsibilities and not having family here. So, when you have kids, you do not have time for yourself. You cannot even go out if you want to. You have to sacrifice many things in life. And you have to focus everything on the baby and your family, so your whole life, you know, it just goes into doing household work like cooking, cleaning, making, feeding and laundry.’

Some women expressed concern about repeated GDM in future pregnancies. Some women reported that their experience of GDM and its management made them more vigilant with diet and exercise in subsequent pregnancies, especially if they also had a family history.

In 8: ‘Yes, it′s for all three pregnancies. I have so many ideas for the other two. The first time there, I was very worried. What to do? What to do if it′s the end of life?’

### 3.5. Preventing T2D

Participants were concerned about the long‐term risk of developing T2D. Although GDM resolves postpartum, it increases the risk of future T2D for women and their children. Some participants were not aware that their child is at risk of being diagnosed with T2D in the long term. Some women believed that ongoing follow‐up was required for themselves and their children to keep them healthy and delay the early onset of T2D.

In 4: ‘No, I wasn′t aware of that. We usually say it′s okay for kids to eat sugar because they are active and digest it while playing and running around. There was not much information provided, and there were no details, especially on how to avoid diabetes in children in case the mother had GD.’

### 3.6. Improving GDM and Postpartum Care

#### 3.6.1. Structured Regular Follow‐Up

Most women reported feeling abandoned soon after postpartum. Most women mentioned cost as a barrier to avoiding post‐GDM visits to their GPs. Participants suggested that to prevent new mothers from moving backwards to unhealthy lifestyles that women had before gestational diabetes, women should receive subsidised structured post‐GDM care soon after delivering a baby and be offered regular follow‐ups, such as blood checks and subsidised appointments with their GP, to monitor blood glucose.

In 12: ‘Yeah, it should be regular. It will be good, and it will give us also some control, like, Okay, I have to go to the GP to get the blood test and make sure to get the sugar under control, because if the result is not good, we again have to go back to all those medicines and treatments.’

#### 3.6.2. Subsidised Consultation With a Dietitian/Nutritionist

All women acknowledged the need to see a dietitian/nutritionist during GDM management and after GDM. They were aware of the high consultation fees and noted that cost is a barrier to accessing such consultations post‐GDM. Participants expressed a need for free or subsidised postnatal follow‐up to provide information on long‐term healthy dietary advice, culturally appropriate food alternatives and recipes that consider traditional cuisines to prevent them from reverting to an unhealthy lifestyle. This could take the form of a consultation with a dietitian or nutritionist, or a helpline.

In 7: **‘**Yeah, especially follow‐up or monitoring, I believe that we need a subsidised consultation or perhaps a one‐time visit to a dietitian, especially a specialist in South Asian diets.’

#### 3.6.3. Access to Advanced Continuous Glucose Monitoring (CGM) Tools

The use of CGM sensors was viewed very positively by those who had tried them; at least three participants reported buying them for themselves to reduce their burden, such as reducing the pain, inconvenience and emotional burden of frequent finger pricks, resulting in improved compliance for those using insulin and a reduction in the financial burden for mothers who fund it themselves. However, because these devices are expensive and not subsidised, participants suggested replacing glucometers with a call for subsidised use of CGM devices (such as Abbott′s Libre and Dexcom) for women with GDM.

In 17: ‘Not government‐funded, and it′s pretty expensive. I think two of them were between $100 and $200. So it′s quite expensive. And I would say it should be subsidised, generally for mums with GD, which made things less overwhelming and less of a job for me.’

#### 3.6.4. Addressing the Emotional Burden

Several participants noted that the emotional toll (stemming from the constant need to monitor and the stigma or shame sometimes attached to being ‘on insulin’ and diabetic) could be reduced through additional support during and post‐GDM, such as better counselling and support groups. Peer support, whether through community‐based programmes or online groups, could help women share strategies for managing stress.

In 10: ‘Mental health is such a big deal, and especially after a baby, because the postpartum period is a very difficult time, and we, especially Asians, do not understand postpartum depression and anxiety, suicidal thoughts and being suicidal. It should be a priority for us as well to have regular mental health sessions.’

#### 3.6.5. Green Prescription (GRx)

Some participants had been referred to a GRx, a NZ primary healthcare initiative in which healthcare professionals provide advice and support to encourage increased physical activity [26]. However, some participants reported difficulty using GRx services because of cultural and religious preferences for gender‐segregated physical activities, such as women‐only swimming sessions, exercise classes or gym facilities. Other participants suggested that advice or online resources supporting home‐based physical activities could help them stay active, even if they cannot use GRx because of childcare and household responsibilities.

In 3: ‘And the barrier for our ethnic ladies is that we will not go to the swimming pool because they are mixed (male and female activity). I also know that some classes have held women‐only swimming days, and perhaps more of these in different areas would be beneficial. Women‐only gym day or something like that, you know.’

#### 3.6.6. Healthcare Equity

Participants who received care in different regions of NZ experienced different care between those regions. Some participants received GDM services from multidisciplinary teams; however, a few participants reported receiving care from a diabetes doctor. This inconsistency was perceived as confusing and unfair. Some participants suggested improved coordination between GPs, midwives and diabetes clinics to ensure that information is consistent across NZ and that regional variation is minimised.

In 20: ‘I had a midwife. But then, she was just for a usual checkup. After I found out I had gestational diabetes, they referred me to the hospital, and then I was dealing with a gestational diabetes doctor.’

## 4. Discussion

Women discussed their experiences and current lifestyles after GDM and offered suggestions for postpartum care. This study identified six themes: awareness and education, culturally appropriate resources, community support, moving backwards to an unhealthy lifestyle, improving postpartum follow‐up and lifestyle transition.

These findings emphasise the need for early counselling for people at risk, culturally tailored resources, consistent follow‐up, family‐inclusive interventions and emotional support, aligning with previous research that advocates for more person‐centred and ongoing postpartum care to prevent the development of T2D [27, 28]. Box 1 summarises the recommendations arising from this work.

Participants highlighted the cultural inadequacy of educational and dietary guidance during and after GDM. This study finds that most participants reverted to their unhealthy lifestyle after GDM, similar to evidence showing that many women follow a healthy lifestyle during pregnancy but fail to maintain it after childbirth [29, 30].

They want dietary resources containing recipes and meal plans that reflect traditional South Asian cuisines, emphasising vegetarian options. This expands on existing literature that emphasises the need for culturally tailored interventions that address and improve dietary adherence, food literacy and long‐term metabolic outcomes within immigrant populations [31]. Educational resources, such as visual aids, can help overcome language barriers and improve accessibility, echoing similar calls in other studies for health education to be both linguistically and culturally appropriate. Participants emphasised that cultural congruence was more important than the ethnicity of the healthcare provider, although some felt South Asian dietitians could offer more contextually relevant advice [31, 32].

The findings highlight a broader gap in the continuity of postpartum care. Participants emphasised the need for regular, subsidised follow‐up consultations—particularly with GPs and dietitians—as well as automated reminder systems, given that cost was identified as a key barrier. Despite existing clinical guidelines recommending postpartum glucose testing, lifestyle counselling implementation remains inconsistent [14, 17]. Our findings echo international evidence suggesting that structured follow‐ups supported by automated reminders and proactive care models improves screening uptake and health behaviour maintenance [33, 34]. Participants′ suggestion of scheduled postpartum reviews reflects a desire for ongoing accountability, guidance and risk monitoring. NZ literature revealed that postpartum T2D screening rates were low and identified significant ethnic and regional disparities across NZ [35].

Psychosocial dimensions were also highlighted. Women felt emotionally burdened by self‐management of GDM and societal stigma around diabetes. This emotional toll is often overlooked in standard GDM care pathways. However, most existing GDM services lack mental health support and cultural support services [33]. Previous studies on ethnic minority groups found cultural beliefs influence disease perception and coping strategies [36]. Participants suggested accessible mental health support, peer‐led groups and culturally safe environments where they could share their experiences and reduce feelings of isolation. Such community‐based interventions show promise in promoting resilience and self‐efficacy [37, 38].

A notable finding was the strong endorsement of CGM tools among women who had access to them. Although cost was a major barrier, CGMs were valued for their ability to reduce the physical discomfort and psychological burden of frequent finger‐pricking. These perceptions are supported by research indicating CGMs improve glycaemic control, patient empowerment and quality of life, especially in high‐risk groups [36]. Subsidised access to such technologies during pregnancy may enhance adherence and outcomes.

Family dynamics emerged as both a facilitator and a barrier to successful GDM management. Participants emphasised the importance of involving husbands and extended family members in education and lifestyle planning. This aligns with previous research showing that health behaviours in South Asian households are often collectively influenced, with older relatives, such as mothers‐in‐law, playing central roles in dietary and caregiving decisions. Without family buy‐in, individual efforts are often undermined, highlighting the importance of designing family‐inclusive interventions [38, 39].

Physical activity was another area where culturally tailored solutions were lacking. Women cited limited access to gender‐segregated exercise spaces, childcare responsibilities and time constraints as key barriers. These findings mirror previous research on physical activity constraints among ethnic minority women [40]. Participants wanted women‐only fitness programmes, community walking groups and home‐based resources tailored to postpartum needs. The limited uptake of GRx further suggests that existing exercise interventions may lack cultural relevance [41], even though a GRx improves both physical and mental health [41].

Women expressed a need for consistency in care and coordination between healthcare providers for GDM across different regions of NZ. Previous literature indicates that recent studies emphasise the need for stronger integration between healthcare providers and diabetes clinics, such as clear referral pathways and consistent messaging. The literature suggested that this could help reduce gaps and improve the overall experience of postpartum care [14, 42]. Our findings support early intervention for people at high risk of GDM. Anticipatory guidance has proven potential to promote healthier pregnancies in reducing the incidence of GDM [43, 44].

**BOX 1: Suggestions for the care of SA women of reproductive age**
● Inform SA women early about their increased risk of GDM and the lifestyle measures that can reduce that risk and prevent GDM and early onset diabetes. Involving husband and family in education and care may enhance adherence to healthy lifestyles.
● Community education for reducing stigma associated with diabetes.
● Develop culturally appropriate educational materials, including relevant advice, dietary resources and nutritional support.
● Consider subsidised access to continuous glucose monitoring for high‐risk women with GDM (prediabetes or GDM more than once) for better management and to prevent the early onset of diabetes.
● Structured long‐term postpartum follow‐up.
● Utilise automated reminder systems through community health centres to prompt women to undergo follow‐up blood tests. Scheduled subsidised follow‐up consultations with GPs, dietitians or other healthcare providers reinforce lifestyle modifications.
● Address the emotional and social impact of GDM through structured peer support, culturally appropriate physical activity options and mental health resources

## 5. Limitations

### 5.1. Strengths and Limitations

This study incorporated suggestions from SA women during the postnatal period, viewing them not just as care recipients but also as active advocates for system improvements after GDM. Their suggestions, based on lived experience, are validated by the research as a key aspect of patient advocacy. The qualitative method yielded rich insights into the cultural, emotional and practical aspects of care that are most important to these women. Notably, the diverse backgrounds within the participant group represent various SA communities, enabling a broader understanding of how healthcare systems can adapt care to meet the different needs of this group. These findings may offer practical guidance to strengthen the tailoring and transferability of future interventions. Notably, the diverse backgrounds within the participant group represented a range of South Asian communities, allowing for a broader understanding of how healthcare systems can adapt care to meet the needs of this population. The study was designed to include participants from multiple South Asian countries and to remain attentive to both shared experiences and differences in perspectives during analysis. However, it was not intended as a formal comparison between countries. In reviewing the data, we did not identify sufficiently consistent or substantial country‐specific variations to warrant reporting separate themes. Instead, participants from different South Asian countries described broadly similar experiences and offered comparable recommendations related to GDM care, postpartum follow‐up, culturally appropriate resources, family involvement and T2D prevention. Given the relatively small numbers within individual country‐of‐birth groups, we considered it more appropriate to emphasise the common themes across the sample. These shared priorities may guide the development of interventions responsive to the needs of diverse South Asian communities while also supporting the broader applicability of findings across similar settings.

Conducting all interviews by telephone offered benefits such as flexible scheduling, less travel and access to participants across NZ. It was also more accessible for participants with childcare, work or transport issues. However, it limited the ability to observe non‐verbal cues and body language, reducing context. Building rapport over the phone could also be harder for some participants. The study included self‐selected participants. Those who chose to engage may have had stronger motivations or more extreme experiences, either positive or negative, than those who declined. Finally, as the data relied on retrospective self‐reporting, recall bias cannot be ruled out.

## 6. Conclusion

This study provides important insights into how culturally appropriate and emotionally supportive care is needed and could improve outcomes for South Asian women and ethnicities at risk of early onset of T2D after GDM. The study identified significant gaps in continuity of care, particularly in postpartum care. Participants advocated for regular follow‐ups and sustained support to address long‐term health risks like T2D. Participants consistently recommended improvements in long‐term postpartum healthcare delivery, emphasising culturally relevant educational materials, mental health integration and tailored dietary resources. Future health system interventions should incorporate these recommendations into their long‐term postpartum care.

## Author Contributions

Sumera Saeed Akhtar: conceptualisation, data curation, formal analysis, funding acquisition, investigation, methodology, resources, software, writing—original draft and writing—review and editing. Sharon Leitch: writing—review and editing.

## Funding

This study was funded by the Health Research Council of New Zealand, 10.13039/501100001505. Open access publishing facilitated by University of Otago, as part of the Wiley ‐ University of Otago agreement via the Council of Australasian University Librarians.

## Disclosure

All authors have read and approved the final version of the manuscript. The corresponding author had full access to all data in this study and takes full responsibility for the integrity of the data and the accuracy of the data analysis.

## Ethics Statement

This project received ethical approval from the University of Otago Human Ethics Committee (Health), under Approval Number 24/004.

## Conflicts of Interest

The authors declare no conflicts of interest.

## Data Availability

The data that support the findings of this study are available on request from the corresponding author. The data are not publicly available due to privacy or ethical restrictions.
